# Early Stroke Rehabilitation of the Upper Limb Assisted with an Electromyography-Driven Neuromuscular Electrical Stimulation-Robotic Arm

**DOI:** 10.3389/fneur.2017.00447

**Published:** 2017-09-04

**Authors:** Qiuyang Qian, Xiaoling Hu, Qian Lai, Stephanie C. Ng, Yongping Zheng, Waisang Poon

**Affiliations:** ^1^Interdisciplinary Division of Biomedical Engineering, The Hong Kong Polytechnic University, Hong Kong, Hong Kong; ^2^Department of Surgery, Prince of Wales Hospital, The Chinese University of Hong Kong, Hong Kong, Hong Kong

**Keywords:** stroke, upper limb, robot, neuromuscular electrical stimulation, electromyography

## Abstract

**Background:**

Effective poststroke motor rehabilitation depends on repeated limb practice with voluntary efforts. An electromyography (EMG)-driven neuromuscular electrical stimulation (NMES)-robot arm was designed for the multi-joint physical training on the elbow, the wrist, and the fingers.

**Objectives:**

To investigate the training effects of the device-assisted approach on subacute stroke patients and to compare the effects with those achieved by the traditional physical treatments.

**Method:**

This study was a pilot randomized controlled trial with a 3-month follow-up. Subacute stroke participants were randomly assigned into two groups, and then received 20-session upper limb training with the EMG-driven NMES-robotic arm (NMES-robot group, *n* = 14) or the time-matched traditional therapy (the control, *n* = 10). For the evaluation of the training effects, clinical assessments including Fugl-Meyer Assessment (FMA), Modified Ashworth Score (MAS), Action Research Arm Test (ARAT), and Function Independence Measurement (FIM) were conducted before, after the rehabilitation training, and 3 months later. Session-by-session EMG parameters in the NMES-robot group, including normalized co-contraction Indexes (CI) and EMG activation level of target muscles, were used to monitor the progress in muscular coordination patterns.

**Results:**

Significant improvements were obtained in FMA (full score and shoulder/elbow), ARAT, and FIM [*P* < 0.001, effect sizes (EFs) > 0.279] for both groups. Significant improvement in FMA wrist/hand was only observed in the NMES-robot group (*P* < 0.001, EFs = 0.435) after the treatments. Significant reduction in MAS wrist was observed in the NMES-robot group after the training (*P* < 0.05, EFs = 0.145) and the effects were maintained for 3 months. MAS scores in the control group were elevated following training (*P* < 0.05, EFs > 0.24), and remained at an elevated level when assessed 3 months later. The EMG parameters indicated a release of muscle co-contraction in the muscle pairs of biceps brachii and flexor carpi radialis and biceps brachii and triceps brachii, as well as a reduction of muscle activation level in the wrist flexor in the NMES-robot group.

**Conclusion:**

The NMES-robot-assisted training was effective for early stroke upper limb rehabilitation and promoted independence in the daily living comparable to the traditional physical therapy. It could achieve higher motor outcomes at the distal joints and more effective release in muscle tones than the traditional therapy.

**Clinical Trial Registration:**

ClinicalTrials.gov, identifier NCT02117089; date of registration: April 10, 2014.

## Introduction

Stroke is one of the leading causes of permanent disability in adults (1). Approximately 80% stroke survivors regain their walking independence (2). However, less than 25% survivors could achieve some limited recovery on the upper limb function, and only around 5% of them could obtain complete functional recovery 6 months later after the onset (2, 3). Dysfunctions in the upper limb are a combination of muscle weakness, spasticity, and discoordination among different muscle groups (4, 5). Significant spontaneous motor recovery usually occurs within the first several weeks to 6 months after stroke, i.e., in the subacute period (6). Physical rehabilitation in this early period can optimize the spontaneous neural plasticity and motor responsiveness, and result in maximized motor outcomes (7, 8). In comparison with the rehabilitation treatment administrated in the chronic period (i.e., 6 months later after the onset), motor functions resorted in the subacute period are more likely to be generalized into functional activities in the daily life (9, 10). One of the major reasons is that the persons with subacute stroke have not been used to adopt the unaffected limb only for daily tasks as commonly observed in the chronic. The traditional rehabilitation treatments in early stage after stroke are usually conducted manually by human therapists, which are time consuming and labor demanding (5). It is challenging to the current medical and health-care system to provide adequate or intensive rehabilitation treatments to persons with subacute stroke, due to the lack of professional manpower in the physical therapy industry even in developed countries (11) and the expanding of stroke populations worldwide (3).

Effective motor restoration after stroke depends on repeated and intensive practice of the paralyzed limbs with voluntary efforts (7, 12, 13). Repetitive practice with high-intensity has been proven to speed up the process of motor restorations (6, 13). The involvement of voluntary effort from the residual neuromuscular pathways has been convinced to carry out better performance with higher efficiency when compared with the continuous passive motion trainings (14, 15). Coordinated upper limb practices among different joints, especially the involvement of the distal joints (e.g., the wrist and fingers) have also been found more effective to translate the motor improvements into meaningful limb functions than single joint practice (16). However, due to the overall muscle weakness in early stage after stroke and a delayed motor return at the distal joints in comparison with the proximal, it is always a difficulty for human therapists to instruct and support the coordinated upper limb motions with the proximal (i.e., the shoulder and the elbow) and distal joints (i.e., the wrist and the fingers) together in the clinical practice (17). New techniques are needed to assist in the manually conducted upper limb coordinating rehabilitation.

Rehabilitation robots can assist human therapists to conduct the intensive and repeated physical training with different numbers and sizes of electrical motors. Various robots have been designed for poststroke upper limb rehabilitation (18–21). Among them, the robots with the involvement of voluntary efforts from the residual neuromuscular pathways demonstrated better rehabilitation effects than those with passive limb motions, i.e., the limb motions are entirely dominated by the machine (18). It has been found that physical trainings with passive motions only contributed to the temporary release of muscle spasticity. However, voluntary practice could improve the motor functions of the limb with longer sustainability (18, 22). Neuromuscular electrical stimulation (NMES) is a technique that can generate limb movements by applying electrical current on the paretic muscles (23). Poststroke rehabilitation assisted with NMES has been found to effectively prevent muscle atrophy and improve muscle strength (23, 24), and the stimulation also evokes sensory feedback to the brain during muscle contraction to facilitate motor relearning (25). NMES can improve limb functions by limiting “learned disuse” that stroke survivors are gradually accustomed to managing their daily activities without using certain muscles, which has been considered as a significant barrier to maximize the recovery (26). However, NMES alone is hard to achieve desired accuracy in kinematics, such as speed and trajectories, as in the robot-assisted training (27).

In our previous works, we designed a series of voluntary intention-driven rehabilitation robotics for physical training at the elbow, the wrist, and fingers (22, 28–31). Residual electromyography (EMG) from the paretic muscles was used to control the robots to provide assistive torques to the limb for desired motions (31, 32). Later, we integrated NMES into the EMG-driven robot as an intact system for wrist rehabilitation. It has been found that the combined assistance with both robot and NMES could reduce the excessive muscular activities at the elbow and improve the muscle activation levels related to the wrist in chronic stroke, which was absent in the pure robot-assisted training (31). Pure robot-assisted upper limb training also showed no superiority on motor improvements on chronic stroke in comparison with the traditional treatments in a reported randomized controlled trial (33). More recently, combined treatment with robot and NMES for the wrist by other research group also demonstrated more promising rehabilitation effectiveness in the upper limb motor recovery than pure robot training (34). However, most of the proposed devices are for single joint treatment, and the related trials were conducted on chronic stroke. We hypothesized that poststroke multi-joint coordinated training with both NMES and robot in the subacute stroke period could improve the muscular coordination in the whole upper limb and translate the motor improvements into daily functions. In this work, we developed an EMG-driven NMES-robotic arm for multi-joint coordinated training on the elbow, wrist, and fingers. The feasibility of the EMG-driven NMES-robotic arm assisted upper limb training on subacute stroke, and the training effectiveness were investigated through a pilot randomized controlled trial in comparison with the traditional upper limb physical rehabilitation.

## Methodology

### The EMG-Driven NMES-Robot System

**Figure 1:**
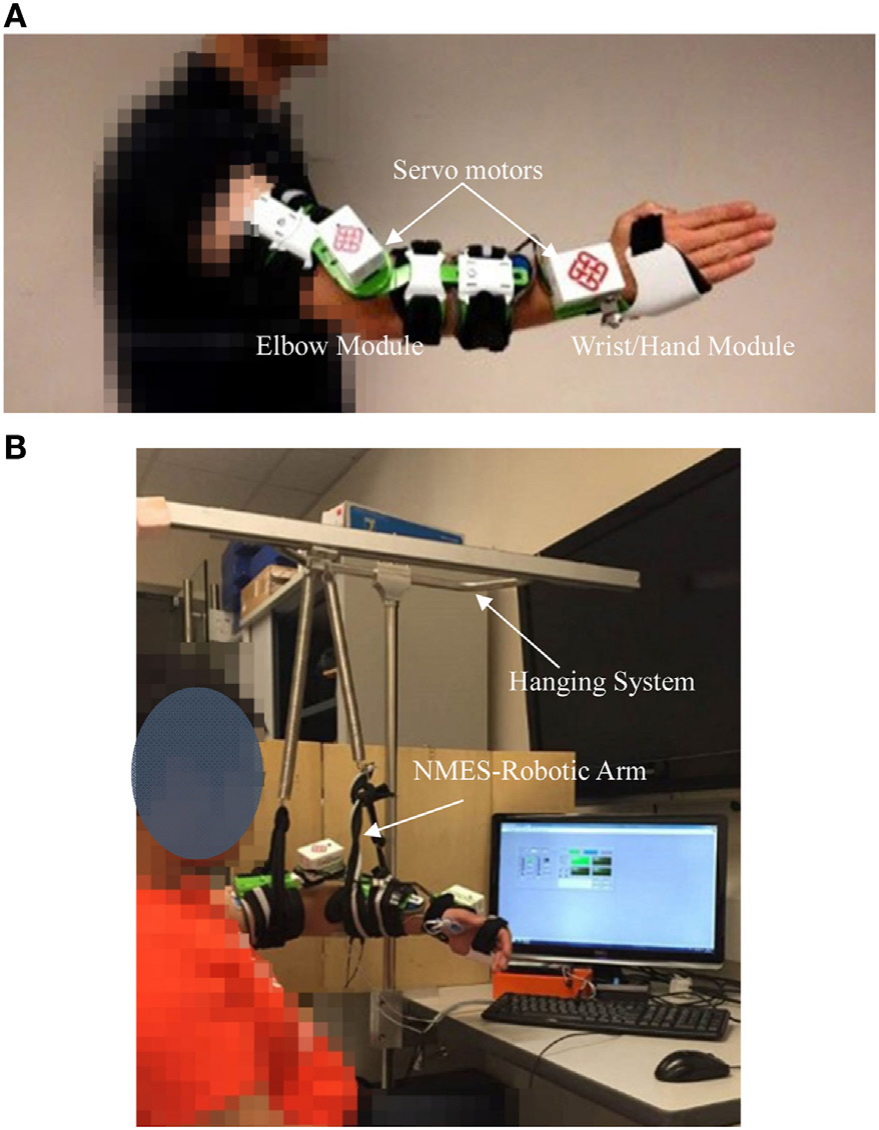
The experimental and training setup of the electromyography-driven neuromuscular electrical stimulation (NMES)-robot system: **(A)** the mechanical exoskeleton module of the system consisted of the wrist part and the elbow part; **(B)** the training setup in a session assisted with the NMES-robot training system, the visual feedback interface, and the hanging system.

The integrated EMG-driven NMES-robot training system (Figure 1) can assist a stroke survivor to perform the sequencing motions, i.e., (1) elbow extension, (2) synchronized wrist extension and hand open, (3) wrist flexion, and (4) elbow flexion, which simulates the coordination of the joints in arm reaching, hand grasping, and withdrawing motions in daily activities. The starting position of the motion cycle was set as elbow joint extended at 180° and the wrist extended at 45°, respectively. The range of motion (ROM) for the elbow joint in the system was set from 30° flexed to 180° extended; and the ROM for the wrist joint was from 60° flexed to 45° extended. The ROMs for the elbow and wrist joints had been tested on their feasibilities when applied to stroke participants in our previous works (27, 28, 31). The paretic arm of a participant could be fixed in a solid exoskeleton orthosis through a bracing system. The movement of the mechanical exoskeleton for the elbow and the wrist parts are controlled by two independent servo motors (MX 106, ROBOTIS, with a maximal stall torque of 8.4 Nm) (27) (Figure 1A). When using the system in this work, the paretic upper limb of a participant mounted with the system was lifted up to a horizontal level with a hanging system (Figure 1B). It was understood that stroke survivors in early stage (e.g., subacute period) usually experienced more muscular weakness rather than spasticity as in the chronic period, and most stroke survivors at this period could not even lift up their paretic limbs with voluntary effort, which was mainly due to the muscle atrophy at the shoulder. The hanging system was necessary for a subacute stroke participant to perform the upper limb tasks with the system in the study.

**Figure 2:**
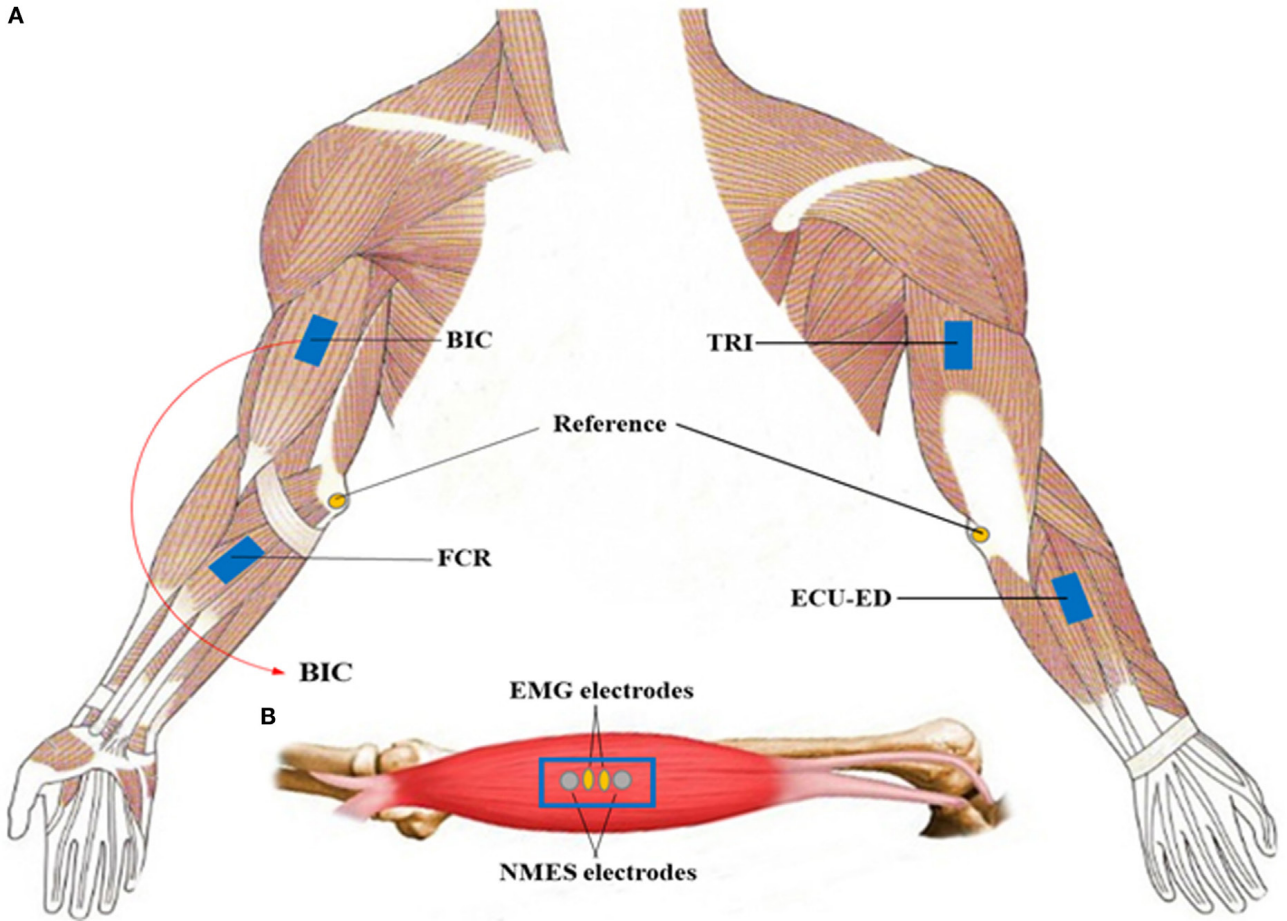
**(A)** The electrode configuration on the target muscles, i.e., the biceps brachii (BIC), the triceps brachii (TRI), flexor carpi radialis (FCR), and the muscle union of the extensor carpi ulnaris and extensor digitorum (ECU-ED). The reference electrode was attached on the olecranon; **(B)** the illustration of the configuration of the electromyography electrodes and neuromuscular electrical stimulation (NMES) electrodes on a target muscle.

Four-channel NMES was applied on the muscles of biceps brachii (BIC) during elbow flexion, triceps brachii (TRI) during elbow extension, flexor carpi radialis (FCR) for wrist flexion, and the last channel on both the extensor carpi ulnaris (ECU) and extensor digitorum (ED) for wrist extension and the associated hand open (i.e., finger extension). The muscles of the ECU and ED are close to each other anatomically with narrow muscle bellies on the dorsal side of the forearm, and can be recruited together by just one-channel surface NMES (35). They were treated as a muscle union (ECU-ED) for both NMES and EMG detection in this work. The function of the motors and NMES were under the control of the EMG detected from the BIC, TRI, FCR, and ECU-ED muscles. The configuration for the EMG and NMES electrodes on a target muscle is shown in Figure 2, which also has been adopted in our previous NMES-robot system for wrist rehabilitation (32). For the ECU-ED muscle union, the EMG and NMES electrodes were located on the common area of the muscle bellies of the two. There was no NMES or robotic support to the hand close motion, since most of the stroke survivors experienced difficulties in hand open rather than hand close (5, 36), and NMES on finger flexors also may accelerate the development of the muscle tones in the fingers (5).

Assistance from both the robot and NMES modules was under the control of EMG signals from the target muscles and helped the participant to conduct the phasic and sequential limb tasks, i.e., (1) elbow extension, (2) wrist extension and hand open, (3) wrist flexion, and (4) elbow flexion. EMG-triggered control was adopted in this work, i.e., in each motion phase, once the EMG activation level of a driving muscle exceeded a preset threshold [three times of the SD above the EMG baseline in the rest, by following the standard detection of the onset of voluntary EMG in a contracting muscle (37)], the related joint motor would move with a constant velocity of 10°/s (either flexion or extension within the ROMs), and it was a joint angular velocity acceptable for stroke survivors in our previous works (31, 32). Meanwhile, constant NMES would also be turned on by the voluntary EMG level which surpassed the triggering threshold and be delivered (square-wave pulses with a constant amplitude of 80 V, stimulation frequency at 40 Hz, individual pulse width at 100 µs) to the target muscle (i.e., the driving muscle in a motion phase) in the related motion phase. Once the joint motors and the NMES were initiated by the EMG signals from the driving muscle, there was no voluntary effort needed from the participant and the training system would help the limb complete the rest of the motion in the phase. All EMG signals were amplified with a gain of 1,000 (amplifier: INA 333, Texas Instruments Inc.), band-pass filtered from 10 to 500 Hz, and then sampled with 1,000 Hz for digitization. The EMG signals during the triggering period for the initiation of the movements were full-wave rectified and moving-averaged with 100 ms window to obtain the EMG activation levels.

### Subject Recruitment and Training Protocol

The study was approved by the Human Subjects Ethics Subcommittee of Hong Kong Polytechnic University and Joint Chinese University of Hong Kong-New Territories East Cluster Clinical Research Ethics Committee. We screened the stroke inpatients in the teaching hospital, and recruited participants with upper limb motor deficits satisfying the following inclusion criteria: (1) had a singular and unilateral brain lesion due to a stroke acquired within 4 months; (2) had standard medical care and sustained in a stable condition; (3) had enough cognition to understand the content or purpose of the study and follow simple instructions as assessed by the Mini-Mental State Examination (MMSE > 21) (38); (4) motor impairments affected in the upper limb ranged from severe to moderate as assessed by Fugl-Meyer Assessment (15 < FMA < 45, with a maximal score of 66 for the upper limb) (39); (5) the spasticity affected at the elbow, the wrist and the fingers below 3 as measured by the Modified Ashworth Scale [MAS, ranged from 0 (no increase in the muscle tone) to 4 (affected part rigid)] (40); (6) the passive ROM of the participants for the wrist was from 45° extension to 60° flexion and the ROM for the elbow was from 30° flexion to 180° extension; (7) aged from 18 to 78 years (41, 42); (8) had detectable voluntary EMG from the target muscles (i.e., three times of the SD above the baseline); (9) had a stable medical condition for physical training with multiple sessions. Subjects were excluded if they did not meet the above inclusion criteria, or had the following conditions: (1) currently pregnant, (2) severe aphasia, and (3) had an implanted pacemaker.

**Figure 3:**
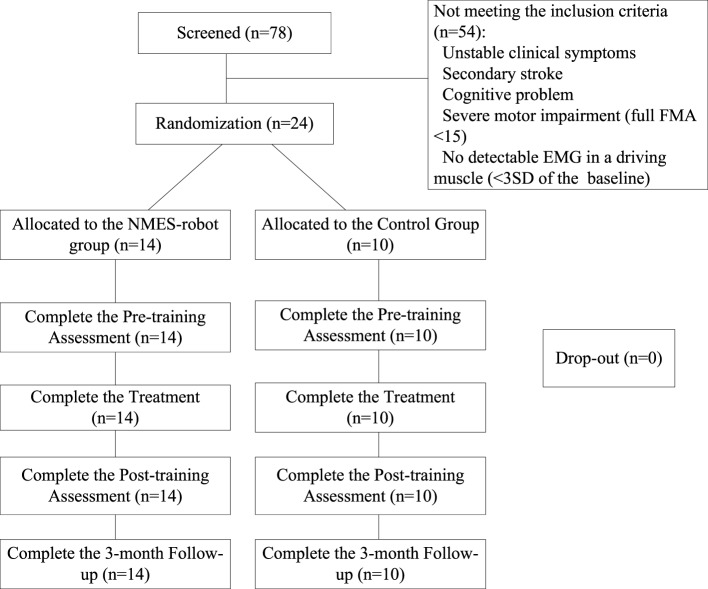
The Consolidated Standards of Reporting Trials flowchart of the experimental design.

The study was a pilot randomized controlled trial with a 3-month follow-up (3MFU). Inpatients after stroke were screened by a collaborative clinician according to the inclusion criteria 7–10 days before the start of the training, in a project period of 24 months. The potential participants were told about the training program of the study, and the recruited participants gave the written consent on the participation in the training program which could be either the device-assisted training or the traditional treatment before the randomization. Then, the recruited participants were randomly assigned into two groups, according to a computer-based random number generator, i.e., the computer program generated either “1” (the experimental group) or “2” (the control group) with an equal probability of 0.5 (Matlab 2015, Mathworks, Inc.). The recruitment of the subjects was in a relatively sequential way due to the availability of the training device (only one set for the respective left and right sides) and the hospital stay of the participants in the management. Once both sides of the robotic arms were occupied for the training, the recruitment was suspended. In the subject screening period, the clinician also needed to take into account of the availability of the device for left or right hemiplegia in the coming possible NMES-robot-assisted training. Figure 3 shows the Consolidated Standards of Reporting Trials flowchart of the training program.

In the experimental group, i.e., the NMES-robot group, each participant received 20-session upper limb training assisted by the NMES-robot system with an intensity of 5 sessions/week, 1 session/day (from Monday to Friday), finished in one month. In each session, a participant was instructed to conduct the device-assisted and repeated limb motions, i.e., (1) elbow extension, (2) wrist extension and hand open, (3) wrist flexion, and (4) elbow flexion for totally 40 min. There was a break of 10 min after a practice of 20 min to avoid the muscle fatigue. In this work, the training loads between the NMES-robot group and the control group were matched by the time and the frequency of the sessions for the upper limb training. The upper limb rehabilitation time of a session for the NMES-robot group was deducted from the routine upper limb training (1 h from Monday to Friday in a common treatment room in the management of the collaborative hospital) by human therapists. The routine upper limb treatment included muscle stretching, passive/assistive ROM and occupational treatments such as feeding/eating, grooming practices. In the practical operation in this work, the participants in the NMES-robot group were transferred to another treatment room to receive the 40-min device-assisted training, and then returned to receive the rest of the routine physical treatments on the upper limb. For most of the participants in the NMES-robot group, they only practiced the muscle stretching and passive ROM after returned from the NMES-robot training for around 10–15 min, due to the fatigue experienced in the upper limb. The participants in the control group only received the routine rehabilitation therapies on the upper limb (i.e., 1 h in the common treatment room).

### Training Effects Evaluation

#### Clinical Assessments

In this study, functional evaluation for each participant on their paretic upper limbs were scored by the following assessments: the FMA (the full score ranging from 0 to 66, the shoulder/elbow part ranging from 0 to 42, and the wrist/hand part ranging from 0 to 24), applied for performance-based measurement of motosensory functions on the poststroke hemiplegia (39); the Action Research Arm Test (ARAT), mainly used to measure one’s hand functions to handle objects in different size, weight and shape (43); the Function Independence Measurement (FIM) for the evaluation of patients’ activities of daily living (ADL) (44); and the MAS, applied for the evaluation of poststroke spasticity at the elbow, the wrist and the fingers (40). All the clinical assessments were conducted before the training started and immediately after the 20 training sessions, as well as 3 months later after the training by a training-blinded assessor. The stroke participants in the study and the assessor were told not to communicate on the training details and the assessor was kept blinded to the training protocol.

#### Session-By-Session Evaluation by EMG

In each training session for the NMES-robot group, a participant was first instructed to perform a bare-arm evaluation task before the device-assisted rehabilitation training, in order to trace the evolution of the muscle coordination during the recovery as we did in the robot-assisted upper limb training on chronic stroke previously (31). The evaluation task had two parts, i.e., horizontal task and vertical task. In the horizontal task, a participant was required to use the affected limb to grasp a sponge and transport it to the lateral side with a distance of 50 cm on a table; then, release the sponge. After that, the participant needed to pick up the sponge again and then transport it back to the original place. In the vertical task, the participant was required to complete the pick-up and release cycle vertically between two layers of a shelf on the table (31, 32). The horizontal and vertical tasks were repeated twice for each with a 5 min break between two consecutive practices to avoid the muscle fatigue. The detailed configuration and description of the two bare-arm evaluation tasks could be found in our previous work (31, 32). The main objective of the bare-arm evaluation in each session is to simulate upper limb motions in daily activities (i.e., hand grasping, arm reaching, and withdrawing) and to reveal the recovery progress across the training sessions of the upper limb motor function without device assistance. In each evaluation task, EMG recording was started when a participant received the command from the trainer, and ended when the testing arm released the sponge at target position. It was understood that subacute patients might not be able to complete the tasks by using their affected limbs independently due to the early muscle weakness in the first several sessions. Therefore, we set a 10-s maximum time limit: if the paretic arm could not grasp the sponge or lift up in 10 s then the participants would be allowed to use the intact hand to help the affected arm grasp or lift up. Only the EMG signals within the 10 s were included for analysis. All participants in the NMES-robot group could grasp the sponge and conduct the horizontal arm transportation with the affected limb in the evaluation from the first session. The major difficulties encountered by the participants were hand open to release the sponge and the lift of the whole arm in the vertical tasks. Therefore, successful hand release of the sponge was not required in this study, although the participants were required to make the voluntary efforts to extend the fingers. The 10-s maximum time limit was mainly applied to the vertical tasks in the early sessions. During the 10-s period, the participants were required to exert voluntary effort to achieve the task by using all possible muscular coordinating strategies in the affected upper limb, with the purpose to record the muscular patterns for an intended target motion. According to our empirical observation, longer attempt periods would cause frustration due to failure and fatigue, i.e., less muscular effects exerted. At the 20th session of the training, all participants recruited in the NMES-robot group could complete the evaluation tasks by the paretic arm without the support from the unaffected limb.

#### EMG Parameters

Two EMG parameters were used for quantitative cross-session monitoring of the muscle activation and coordination pattern changes during the evaluation in this work: (1) normalized EMG activation level of each muscle; and (2) normalized co-contraction index (CI) between the muscle pairs. The processing methods of the normalized EMG activation level was calculated as follows, i.e.,

(1) $$\bar{\text{EMG}}=\frac{1}{T}\int_{0}^{T}{\text{EMG}}_{i}\left(t\right)dt,$$

where $\bar{\text{EMG}}$ was the EMG activation level of muscle *i*, EMG*_i_*(*t*) was the EMG linear envelope with respect to the maximal value recorded during the bare-arm evaluation tasks and maximum voluntary contractions in each session, and *T* was the length of the signal as we did previously (22, 28). In this work, the EMG activation levels in a session for an individual participant were further normalized with respect to the maximal EMG activation level of the participant recorded across the training sessions. This operation would show the tendency of the EMG activation level of a participant across the training session with the normalized values vary from 0 to 1, in order to minimize the variations among different participants as we encountered previously (22, 28).

The CI between a pair of muscles could be expressed as:

(2) $$\text{CI}=\frac{1}{T}\int_{0}^{T}A_{ij}\left(t\right)\mathrm{dt},$$

where *A_ij_*(*t*) represented the overlapping activity (i.e., *Minimum*[EMG*_i_*(*t*), EMG*_j_*(*t*)]) of the EMG linear envelopes for muscle *i* and *j*, and *T* was the length of the signal, EMG_i,j_(t) are the EMG envelopes as in Eq. 1 (22, 28). An increase of the CI values would represent an enlarged co-contraction phase of a muscle pair; and a decrease would suggest a separation in the co-contraction phase of the two muscles within the same joint or across multi joints. Similar normalization on the CI values in a session with respect to the maximal CI value across the sessions for individual participants was conducted as we did for the EMG activation levels. Monitoring the varying patterns of the EMG parameters across the 20 training sessions would provide a better understanding on the recovery progress of the affected upper limb functions.

### Statistical Analysis

The baselines of the two groups were first compared by independent *t*-test with an insignificant statistical difference (*P* > 0.05) on all clinical assessments (i.e., pre-assessments on FMA, MAS, ARAT, and FIM scores, Table 2) to ensure the likelihood of the baseline equivalence (45). Two-way analysis of covariance (ANCOVA) was then used to evaluate the differences with respect to the independent factors of the group (i.e., the NMES-robot and the control groups) and the time point on the clinical assessments (i.e., the pre-, the post-, and the 3MFU assessments) by taking the pre-assessment as a covariate, with the purpose to further minimize the possible baseline difference between the groups (45). Then, one-way analysis of variance (ANOVA) was conducted to investigate the intragroup difference of either NMES-robot group or control group at different time points with the Bonferroni *post hoc* tests. The *post hoc* between-group comparisons on the clinical scores at the respective post- and 3MFU assessments were evaluated by one-way ANCOVA with the pre-assessment as a covariate. The EMG parameters (i.e., EMG levels and CI values) across the 20 sessions were also analyzed by one-way ANOVA for the investigation of recovery process across the whole training sessions in the NMES-robot group. The primary outcomes of the study were the FMA and MAS clinical scores; and the other clinical scores and EMG parameters were the secondary outcomes. It was because FMA could reflect task-specified voluntary motor functions in the whole upper limb and MAS could reflect the variation of muscle spasticity at different joints in the upper limb compared to other clinical scores. The levels of statistical significance were indicated at 0.05, 0.01, and 0.001 in this study.

## Results

We screened 78 stroke inpatients in the wards of the hospital, and 54 of them did not meet the inclusion criteria with single or multiple reasons of (1) unstable clinical symptoms for continuous and long-term physical training, (2) secondary stroke, (3) cognitive impairment, (4) severe motor impairment (full FMA < 15), and (5) no detectable EMG in a driving muscle (<3 SD of the baseline). A total of 24 participants fulfilled the inclusion criteria and were recruited in this study. They were randomly assigned into two groups: the NMES-robot group (*n* = 14) and the control group (*n* = 10). The demographic data of the participants after the randomization are presented in Table 1.

**Table 1:** Demographic data of the participants after the randomization.

Group	No. of participants	Min/Max days after stroke	Stroke types, hemorrhage/ischemic	Lesion site, left/right	Gender, female/male	Age (years)
Neuromuscular Electrical Stimulation (NMES)-robot	14	25/148	9/5	11/3	5/9	54.6 ± 11.3
Control	10	14/142	6/4	9/1	4/6	64.6 ± 3.43

**Figure 4:**
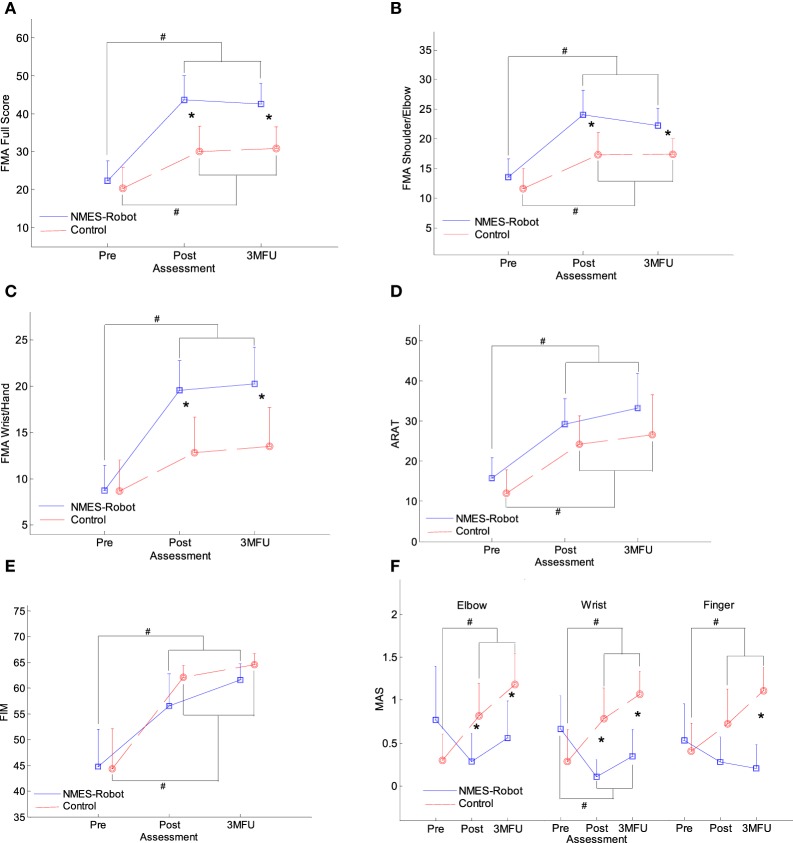
The clinical scores [evaluated before the first and after the 20th training session, as well as the 3-month follow-up (3MFU)] of the participants in both neuromuscular electrical stimulation (NMES)-robot and control groups: **(A)** Fugl-Meyer Assessment (FMA) full scores, **(B)** FMA shoulder/elbow scores, **(C)** FMA wrist/hand scores, **(D)** Action Research Arm Test (ARAT) scores, **(E)** Function Independence Measurement (FIM), and **(F)** Modified Ashworth Score (MAS) scores at the elbow, the wrist, and the fingers, presented as mean value with 2-time SE (error bar) in each evaluation session. The solid lines are for the NMES-robot group, and the dashed lines are for the control group. The significant inter-group difference is indicated by “*” (*P* < 0.05, one-way analysis of covariance), and “#” is used to indicate the significant intragroup difference (*P* < 0.05, one-way analysis of variance with Bonferroni *post hoc* tests).

Figure 4 presents the clinical scores of participants in both the NMES-robot and control groups, with FMA, ARAT, FIM, and MAS evaluated at three time points: before the first training session (pre-training assessment), immediately after the last (20th) training session (post-training assessment), and 3 months after the last training session (i.e., 3MFU). Table 2 summarizes the means and 95% confidence intervals of each clinical assessment together with the two-way ANCOVA probabilities and the estimated effect sizes (EFs) with respect to session and group, and the one-way ANOVA probabilities with the EFs for the intragroup evaluation with respect to the assessment sessions. Table 3 shows the probabilities and EFs of the between-group comparison on the respective post- and 3MFU assessments by one-way ANCOVA with the adjustment of the baseline effect.

**Table 2:** The mean and 95% confidence intervals for each measurement of the clinical assessments, and the probabilities with the estimated effect sizes of the statistical analyses.

	Pre	Post	3MFU	1-way ANOVA	2-way analysis of covariance		
					*P* (Partial η^2^)		
			
Evaluation	Mean (95% Confidence Interval)			*P* (Partial η^2^)	Session	Group	S*G
**FMA**							
Full score, Neuromuscular Electrical Stimulation (NMES)-robot	22.3 (16.5–28.1)	43.6 (37.9–49.4)	42.5 (36.7–48.3)	0.001^###^ (0.475)	0.000^ΔΔΔ^ (0.615)	0.000^ΔΔΔ^ (0.282)	0.003^ΔΔ^ (0.160)
Control	20.3 (14.2–26.4)	30.1 (24.0–36.2)	30.9 (24.8–37.0)	0.031^#^ (0.227)			
Shoulder/elbow, NMES-robot	13.6 (10.1–17.0)	24.1 (20.6–27.5)	22.3 (18.9–25.7)	0.000^###^ (0.360)	0.000^ΔΔΔ^ (0.401)	0.000^ΔΔΔ^ (0.112)	0.029^Δ^ (0.047)
Control	11.6 (8.2–15.0)	17.3 (13.9–20.7)	17.4 (14.0–20.8)	0.030^#^ (0.229)			
Wrist/hand, NMES-robot	8.7 (5.3–12.1)	19.6 (16.2–22.9)	20.2 (16.8–23.6)	0.000^###^ (0.435)	0.000^ΔΔΔ^ (0.551)	0.000^ΔΔΔ^ (0.311)	0.001^ΔΔΔ^ (0.184)
Control	8.7 (4.8–12.6)	12.8 (8.9–16.7)	13.5 (9.6–17.4)	0.176 (0.021)			
**ARAT**							
NMES-robot	15.7 (8.8–22.6)	29.2 (22.3–36.1)	33.2 (26.3–40.1)	0.002^##^ (0.268)	0.000^ΔΔΔ^ (0.279)	0.284 (0.018)	0.912 (0.003)
Control	12.0 (4.0–20.0)	24.2 (16.2–32.2)	26.6 (18.6–34.6)	0.030^#^ (0.229)			
**FIM**							
NMES-robot	44.7 (38.8–50.6)	56.6 (50.7–62.5)	61.6 (55.7–67.5)	0.001^###^ (0.311)	0.000^ΔΔΔ^ (0.542)	0.117 (0.037)	0.418 (0.027)
Control	44.3 (39.3–49.3)	62.1 (57.1–67.1)	64.6 (59.6–69.6)	0.000^###^ (0.603)			
**MAS**							
Elbow, NMES-robot	0.8 (0.3–1.3)	0.3 (−0.2–0.8)	0.6 (0.1–1.0)	0.362 (0.051)	0.051 (0.087)	0.000^ΔΔΔ^ (0.204)	0.001^ΔΔΔ^ (0.201)
Control	0.3 (−0.1–0.7)	0.8 (0.5–1.2)	1.2 (0.8–1.5)	0.005^##^ (0.322)			
Wrist, NMES-robot	0.7 (0.3–1.0)	0.1 (−0.2–0.4)	0.3 (0.0–0.7)	0.048^#^ (0.145)	0.119 (0.064)	0.000^ΔΔΔ^ (0.232)	0.000^ΔΔΔ^ (0.241)
Control	0.3 (−0.1–0.6)	0.8 (0.4–1.1)	1.1 (0.7–1.4)	0.009^##^ (0.292)			
Finger, NMES-robot	0.5 (0.2–0.9)	0.3 (−0.1–0.6)	0.2 (−0.1–0.5)	0.354 (0.052)	0.425 (0.026)	0.000^ΔΔΔ^ (0.176)	0.005^ΔΔ^ (0.152)
Control	0.4 (0.1–0.7)	0.7 (0.4–1.1)	1.1 (0.8–1.4)	0.025^#^ (0.240)			

Differences with statistical significance are marked with superscripts beside the *P* values (“#” for 1-way-ANOVA intragroup tests, “Δ” for 2-way ANCOVA tests on the group and session effects with the pre-assessment as the covariate). Significant levels are indicated as, 1 superscript for <0.05, 2 superscripts for ≤0.01, and 3 superscripts for ≤0.001. The degrees of freedom in the 1-way-ANOVA tests are (1) NMES-robot group, the corrected total = 41, between-group = 2, and within-group = 39; (2) the control group, the corrected total = 29, between-group = 2 and within-group = 27. The degrees of freedom in the 2-way ANCOVA tests are (1) the corrected total = 71, (2) pre-test = 1, (3) Session = 2, (4) Group = 1, and (5) S*G = 67.

FMA, Fugl-Meyer Assessment; MAS, Modified Ashworth Score; ARAT, Action Research Arm Test; FIM, Functional Independence Measurement; 3MFU, 3-month follow-up; ANOVA, analysis of variance; S*G, the interaction between the session and group.

**Table 3:** The statistical probabilities and the estimated effect sizes of the 1-way analysis of covariance (ANCOVA) on the respective post-assessment and 3-month follow-up (3MFU) between the groups, by taking the pre-assessment as the covariate.

Evaluation	1-way ANCOVA on the Post- and 3MFU assessments between the groups	
	Post__Pre_ *P* (Partial η^2^)	3MFU__Pre_ *P* (Partial η^2^)
**FMA**		
Full score	0.000*** (0.478)	0.005** (0.319)
Shoulder/elbow	0.037* (0.190)	0.040* (0.186)
Wrist/hand	0.000*** (0.538)	0.005** (0.322)
Action Research Arm Test (ARAT)	0.417 (0.032)	0.455 (0.027)
Function Independence Measurement (FIM)	0.123 (0.109)	0.169 (0.088)
Modified Ashworth Score (MAS)		
Elbow	0.003** (0.359)	0.004** (0.334)
Wrist	0.001*** (0.430)	0.002** (0.367)
Finger	0.074 (0.144)	0.000*** (0.507)

Differences with statistical significance are marked with ‘*’ beside the *P* values. Significant levels are indicated as, * for <0.05, ** for ≤0.01, *** for ≤0.001. The degrees of freedom are (1) the corrected total = 23, (2) pre-test = 1, (3) between-group = 1, and (4) within-group = 21.

Figures 4A–C show the variation in FMA scores at the three evaluation sessions. Significant differences were observed with respect to the factors of group and session in the FMA full score, the FMA shoulder/elbow and FMA wrist/hand sub-scores (two-way ANCOVA, *P* < 0.05, Table 2). The interactions between the group and session factors were also statistically significant for the three FMA scores (*P* < 0.05, Table 2), where the FMA wrist/hand achieved the most significant level (*P* = 0.001, EF = 0.184, Table 2) and the FMA shoulder/elbow achieved the least significant level (*P* = 0.029, EF = 0.047, Table 2). For the FMA full score (Figure 4A), both groups demonstrated significant increases immediately after the training (i.e., post-assessment), and these increments with respect to the pre-assessment were maintained 3 months later after the training (*P* < 0.05, one-way ANOVA with *post hoc* tests, Table 2). The increments in the FMA full score for the NMES-robot group were significantly higher than the control group at the post- and 3MFU assessments (one-way ANCOVA, *P* < 0.01, Table 3). In Figures 4B,C, the FMA shoulder/elbow and wrist/hand scores demonstrated the similar behaviors as those observed in the FMA full score. However, the FMA wrist/hand scores indicated more significant levels with larger EFs in the interaction between the group and session (two-way ANCOVA, Table 2), and in the between-group one-way ANCOVA comparisons at the post- and 3MFU assessments (Table 3). There was no significant improvement in the FMA wrist/hand score for the control group after the training (*P* > 0.05, one-way ANOVA, Table 2).

Figure 4D presents the ARAT scores in the pre-training assessment, post-training assessment, and 3MFU for both groups. Significant difference was observed with respect to the evaluation sessions (*P* < 0.001, EF = 0.279, two-way ANCOVA, Table 2), whereas no significant difference was observed with respect to the groups. The ARAT scores significantly increased after training in both the NMES-robot and the control groups (*P* < 0.05, one-way ANOVA with Bonferroni *post hoc* tests), and the improvement could be maintained for 3 months (*P* < 0.05, one-way ANOVA with Bonferroni *post hoc* tests).

Function Independence Measurement scores in both the NMES-robot and control groups are shown in Figure 4E. Significant difference was observed with respect to the factor of the evaluation time points (*P* < 0.001, EF = 0.542 two-way ANCOVA, Table 2), whereas no significant difference was observed with respect to the factor of the groups. The FIM scores were significantly higher in the post-training assessment and 3MFU compared with those in the pre-training assessment for both groups (*P* ≤ 0.001, one-way ANOVA with Bonferroni *post hoc* test).

Figure 4F displays the variation in MAS scores at the finger, wrist, and elbow across the evaluation sessions for the two groups. Significant group differences were detected by two-way ANCOVA (*P* < 0.001, EF > 0.176, Table 2). Significant interactions between the factors of the group and the evaluation time point were also captured at all three parts (i.e., elbow, wrist, and fingers) (*P* < 0.01, EF > 0.152, Table 2). The MAS scores were significantly elevated at the elbow, wrist, and fingers at the post-assessment in the control group and were remained above the elevated levels when assessed 3 months later (*P* < 0.05, one-way ANOVA with Bonferroni *post hoc* tests, Table 2). Significant decrease in MAS was observed at the wrist for the NMES-robot group, and this decreased level was maintained as detected by the 3MFU assessment (one-way ANOVA, *P* = 0.048, EF = 0.145, Table 2). There was no significant variation in the elbow and finger MAS scores for the NMES-robot group (*P* > 0.05, one-way ANOVA, Table 2). In the between-group comparison on the MAS, significant lower MAS scores were observed in the NMES-robot group at the elbow and the wrist during the post-assessment (*P* < 0.01, EF > 0.359, one-way ANCOVA, Table 3), and at all joints during the 3MFU assessment (*P* < 0.01, EF > 0.334, one-way ANCOVA, Table 3).

**Figure 5:**
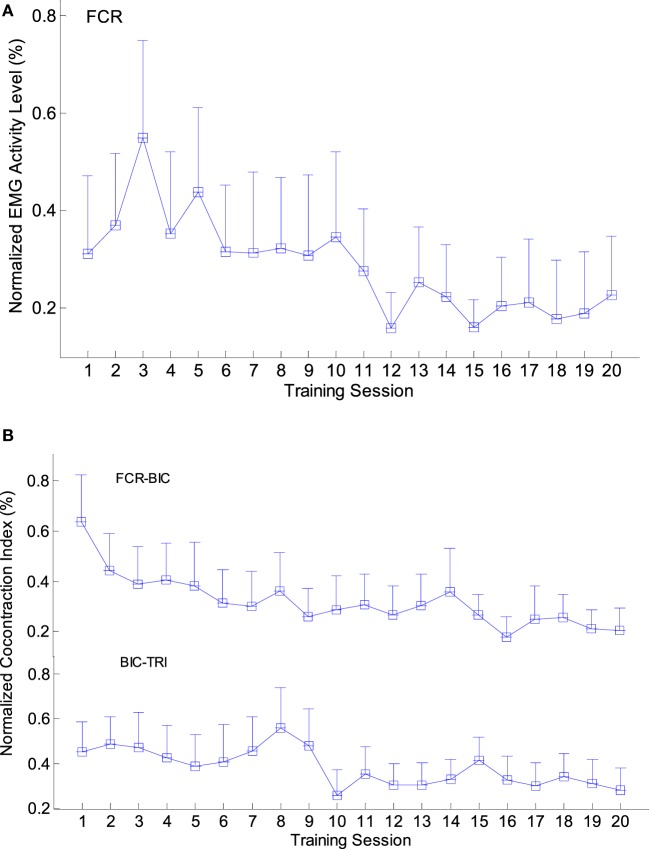
The variation of electromyography (EMG) parameters recorded across the 20 training sessions: **(A)** the changes of the normalized EMG activation levels with significant decline observed in the flexor carpi radialis (FCR) muscle (*P* < 0.05, 1-way analysis of variance (ANOVA) with Bonferroni *post hoc* tests); **(B)** the significant decline of the normalized co-contraction Indexes (CI) values observed in the BIC&TRI and FCR&BIC muscle pairs (*P* < 0.05, 1-way ANOVA with Bonferroni *post hoc* tests). The values are presented as mean value with 2-time SE (error bar) in each session.

Figure 5 shows the variation patterns of EMG parameters (i.e., EMG activation level and CI) across the 20 training sessions in the NMES-robot group. A significant reduction was observed in the EMG activation levels of the FCR (Figure 5A; *P* < 0.05, one-way ANOVA with Bonferroni *post hoc* tests). The EMG activation levels increased in the first few training sessions and reached the peak around the third session. Subsequently, the values decreased in the following 17 sessions and finally reached a plateau in the last five training sessions. No significant variation was observed in other target muscles (BIC, TRI, and ECU-ED). Figure 5B presents the variation patterns of CI values among different muscle pairs either within a single joint or across multiple joints. The CI values of the FCR&BIC and BIC&TRI muscle pairs were significantly reduced along the 20 training sessions (*P* < 0.05, one-way ANOVA with Bonferroni *post hoc* test). The CI values of both muscle pairs reached the peak within the first eight training sessions and then continually decreased in the following process, then reached a steady level in the last three sessions. No significant change was observed in CI values of other muscle pairs.

## Discussion

The results of this study indicated that in the early stage after stroke, motor function in the paretic upper limbs of stroke participants could be significantly improved through both traditional rehabilitation treatment and upper limb training by the EMG-driven NMES-robotic system. The ARAT and FIM scores suggested that the effects of early intervention using EMG-driven NMES-robot training were comparable to the effects of traditional treatments; these findings reflected the improved upper limb function, particularly, in the hand (by ARAT), and improved independence of ADL (by FIM) observed in this study. Although no specific tasks were assigned to the finger joints in this study, the increase in the ARAT scores after NMES-robot training also indicated the voluntary motor improvement in the fingers after the treatments. We noticed that there was no significant improvement in the FIM scores observed after NMES-robot training for chronic stroke patients in our previous study (31). However, in this study, the FIM score results suggested that NMES-robot-assisted intervention was effective in enhancing stroke patients’ ADL levels in the early stage after stroke. Moreover, these improvements could last till 3 months later.

As indicated by the FMA scores, the NMES-robotic arm developed in this study could assist stroke patients to improve the motor function of their entire paretic upper limb, with voluntary effort. Both the NMES-robot training and traditional physical therapies improved the participants’ motor function at the shoulder and elbow, and this improvement was maintained even after 3 months. However, the NMES-robot group achieved greater improvement in the FMA shoulder/elbow and wrist/hand scores than did the control group. Two possible reasons for the improvements in the entire upper limb in the NMES-robot group, especially the shoulder (no actuated assistance in the training), motor function are as follows: (1) The participants’ shoulder-related muscles for arm lifting were practiced during the training process supported by the hanging system used in this study and (2) when the muscle around a joint was trained, the adjacent proximal joint would be simultaneously improved, as indicated in our previous study (31); accordingly, wrist training led to improved elbow function in our previous study, and elbow training led to shoulder improvement in this study. We also observed that through the EMG-driven NMES-robot-assisted training, participants achieved significant motor improvement in the wrist/hand, as indicated by their FMA scores, whereas no significant improvement was achieved in the participants of the control group, who were given traditional physical treatments. The possible reason could be that compared with the rehabilitation of the proximal joints (e.g., shoulder and elbow), motor recovery in the distal joints is more difficult to manage in the manual rehabilitation. Usually, it is hard for a therapist to manage distal and proximal joint training at the same time in the physical practice; hence, most of the manual-conducted training regimens follow a proximal-to-distal sequence (46, 47). Besides, in the early phase after stroke, manually provided physical trainings are focused on the proximal joints, and little effort is allocated for the distal joints (26, 48). Although the movements provided by the NMES-robot system for a joint were simple flexion and extension, the target joints could be more precisely exercised with the aid of well-positioned motors and NMES, and could be organized into multi-joint coordinated movements with computer programs. In addition, traditional manual therapies alone usually cannot be used to exert voluntary effort in the wrist extensors (i.e., ECU-ED in this study) in the early phase after stroke (48). By contrast, NMES-robot training could be used to apply physical training directly to the wrist joint and to coordinate motor practice across multiple joints synchronously, with mechanical support from the servo motors at the wrist and elbow. The assistance from NMES could produce repetitive motosensory experiences and enable the participants to concentrate more on the target muscles at the distal joints, thus helping to evoke the voluntary effort (49, 50).

After the traditional rehabilitation treatments, the participants’ muscle tone (spasticity) increased significantly at all three parts (elbow, wrist, and finger joints). This could be because of the following: (1) the muscle tone was gradually generated through spontaneous recovery process, following the pathological sequence after stroke (51); (2) compensatory muscular activity increased due to the fatigue during the motor practices (31); and (3) motor stimulation in the flexors increased during the traditional physical training process, with a lack of synchronous spasticity control. Although the changes in MAS scores in the NMES-robot group were not significant for the elbow and fingers, the results of interactions (S*G) (Table 2) revealed a completely different evolutionary trend in the muscle tone compared with the control group. The muscle tone declined at all three parts (elbow, wrist, and finger joints) in the NMES-robot group, and a significant reduction was observed at the wrist, which suggested that NMES-robot training could effectively release the muscle spasticity at the wrist joint, and this effect could be maintained until 3 months later. One of the possible reasons could be the intensive practice in a short period for the NMES-robot group in this work. The frequency of the EMG-driven NMES-robot arm assisted training (5 sessions/week, finished in one month) was higher than other clinical trials with equivalent total training hours practiced manually, for example, 3 times/week and finished in 16 weeks (52), where between-group differences could be submerged by other baseline effects. A significant release of muscle spasticity at the finger joints was also observed in our previous study on chronic stroke when NMES and robotic assistance were provided to the wrist joint with a high training frequency (31). In this sense, NMES-robot-assisted upper limb rehabilitation could be a relatively affordable complement to the traditional manual rehabilitation, without too much additional manpower due to the automation.

The improvement in upper limb motor function in the NMES-robot group was reflected by the clinical scores, and the session-by-session recovery progress was revealed through the EMG parameters. The reduced EMG activation levels of FCR implied a release of muscle spasticity at the wrist, which was consistent with the variation in the MAS wrist scores. Most of the patients reached a steady state after the 15th training session. The reduction of FCR was also related to the decrease in the CI values of FCR&BIC, indicating a release in the co-contraction patterns between the elbow and wrist joints. These joints could be moved more independently during arm withdrawing/flexing motions. In addition, a significant reduction in CI values between the BIC&TRI muscle pair was observed, suggesting improved coordination between the flexors and extensors at the elbow joint and an improved independence in muscle contraction over the 20 training sessions. The EMG activation levels of the FCR increased in the first 3–4 training sessions, and the CI values of BIC&TRI muscle pairs reached the peak within the first eighth training session. This was reasonable because in subacute stroke, most of the patients experience muscle weakness in the very beginning, and the muscle strength then recovers through both spontaneous processes and physical training. In addition, the participants needed to adapt to the training process in the first several training sessions. The results of the EMG parameters suggested that NMES-robot training could help release the muscle spasticity and promote muscle coordination within and across different joints effectively, particularly at the wrist in this work.

## Limitation

The main limitation of this study is the small sample size. Despite the relatively small populations recruited, we observed consistent results on the motor improvements achieved in the NMES-robot group by clinical assessments and EMG parameters. The EMG parameters were only recorded for the NMES-robot group to provide the understanding on the evolutionary process of the muscular activities under the training program. Unbalanced arms were obtained after the randomization in this work, mainly due to the limited project period and the small sample size recruited in a relatively sequential way. Randomized clinical trials with larger scales (e.g., larger sample sizes and multi-centers) will be conducted to consolidate the rehabilitation effectiveness of the EMG-driven NMES-robot-assisted upper limb training in the future.

## Conclusion

In this work, the EMG-driven NMES-robot arm was applied for multi-joint coordinated upper limb rehabilitation on subacute stroke participants in comparison with the traditional physical therapy. Both of the treatments could significantly promote the independence in daily activities with comparable intensities. The NMES-robot-assisted training could be more effective in releasing muscle tones and in improving the muscle coordination in the whole upper limb, because of the intensive practices on both the proximal and the distal joints delivered in a short period of rehabilitation. All the training effects achieved by the NMES-robot-assisted rehabilitation could be maintained for 3 months after the training. The NMES-robot-assisted upper limb training could be complementary to the traditional manual training to cope with the shortage of the human rehabilitation professionals in the industry.

## Ethics Statement

The study was carried out in accordance with the human ethic guidelines of the Human Subjects Ethics Subcommittee of Hong Kong Polytechnic University and Joint Chinese University of Hong Kong-New Territories East Cluster Clinical Research Ethics Committee. All participants recruited in the study gave written informed consent in accordance with the Declaration of Helsinki before the start of the training. The medical status of the participants was monitored by the routine management in the hospital.

## Author Contributions

QQ and QL contributed in the NMES-robot arm assisted training experiment, data collection and analysis, and manuscript drafting. SN and WP contributed in the clinical trial design and subject management. YZ contributed in the data analysis. XH conceived of the study and coordinated the whole project, including the clinical trial design, human subject experiments, and manuscript drafting.

## Conflict of Interest Statement

The authors declare that the research was conducted in the absence of any commercial or financial relationships that could be construed as a potential conflict of interest.
